# Synergistic effect and ultrastructural changes in *Trypanosoma cruzi* caused by isoobtusilactone A in short exposure of time

**DOI:** 10.1371/journal.pone.0245882

**Published:** 2021-01-28

**Authors:** Júlio Menta de Almeida, Felipe Oliveira Nunes, Lígia Fernanda Ceole, Tabata D’Maiella Freitas Klimeck, Letícia Alves da Cruz, Danilo Tófoli, Beatriz Santana Borges, Walmir Silva Garcez, Inês Aparecida Tozetti, Lia Carolina Soares Medeiros, Fernanda Rodrigues Garcez, Alda Maria Teixeira Ferreira

**Affiliations:** 1 Laboratório de Imunologia, Biologia Molecular e Bioensaios do Instituto de Biociências, Universidade Federal de Mato Grosso do Sul, Campo Grande, MS, Brazil; 2 Laboratório de Pesquisa de Produtos Naturais Bioativos do Instituto de Química, Universidade Federal de Mato Grosso do Sul, Campo Grande, MS, Brazil; 3 Laboratório de Biologia Celular, Instituto Carlos Chagas (Fiocruz-Paraná), Curitiba, PR, Brazil; Government College University Faisalabad, PAKISTAN

## Abstract

Butanolides have shown a variety of biological effects including anti-inflammatory, antibacterial, and antiprotozoal effects against certain strains of *Trypanosoma cruzi*. Considering the lack of an effective drug to treat *T*. *cruzi* infections and the prominent results obtained in literature with this class of lactones, we investigated the anti-*T*. *cruzi* activity of five butanolides isolated from two species of Lauraceae, *Aiouea trinervis* and *Mezilaurus crassiramea*. Initially, the activity of these compounds was evaluated on epimastigote forms of the parasite, after a treatment period of 4 h, followed by testing on amastigotes, trypomastigotes, and mammalian cells. Next, the synergistic effect of active butanolides against amastigotes was evaluated. Further, metacyclogenesis inhibition and infectivity assays were performed for the most active compound, followed by ultrastructural analysis of the treated amastigotes and trypomastigotes. Among the five butanolides studied, majoranolide and isoobtusilactone A were active against all forms of the parasite, with good selectivity indexes in Vero cells. Both butanolides were more active than the control drug against trypomastigote and epimastigote forms and also had a synergic effect on amastigotes. The most active compound, isoobtusilactone A, which showed activity against all tested strains inhibited metacyclogenesis and infection of new host cells. In addition, ultrastructural analysis revealed that this butanolide caused extensive damage to the mitochondria of both amastigotes and trypomastigotes, resulting in severe morphological changes in the infective forms of the parasite. Altogether, our results highlight the potential of butanolides against the etiologic agent of Chagas disease and the relevance of isoobtusilactone A as a strong anti-*T*. *cruzi* drug, affecting different events of the life cycle and all evolutionary forms of parasite after a short period of exposure.

## Introduction

Chagas disease, caused by the protozoan parasite *Trypanosoma cruzi*, is still a major public health concern worldwide, and is associated with many secondary diseases, disability, early retirement, and extensive treatment costs [1].

Considering the lack of an appropriate drug for treatment of *T*. *cruzi* infection during the chronic phase of the disease, the development of a new and more effective anti-*T*. *cruzi* drug could mitigate the economic, social, and health problems related to this disease [2].

Worsening this scenario, oral infection has exceeded the classic transmission, and has become the foremost common infection in recent years in Brazil. This type of transmission leads to a more aggressive infection, as studies have shown that more than a quarter of the patients could succumb at acute phase due to cardiac complications, which highlights the importance of immediate treatment [3–5].

Lactones have shown various biological activities including anti-inflammatory, antibacterial, and antiprotozoal effects [6–8]. Previous studies have reported that polyketide-derived γ-lactones bearing long α-alkylidene side chains have inhibitory activity against Y strain [9] and Dm28c strain [10] of *T*. *cruzi*, which belong to the discrete typing units (DTUs) TcII and TcI, respectively. In previous study, we isolated and characterized three butanolides (epilitsenolide C1 (**1**), epilitsenolide C2 (**2**) and isoobtusilactone A (**3**)) from the active extract of the xylopodium of *Aiouea trinervis* Meisn. (Lauraceae), among which, isoobtusilactone A and epilitsenolide C1 showed activity against epimastigote forms *in vitro*, with low cytotoxicity and high selectivity index. Isoobtusilactone A was also able to inhibit the growth of amastigote forms in more than 70% after 24 h of treatment [10].

Other butanolides, namely majoranolide (**4**) and rubrenolide (**5**), were obtained from the leaves and fruits of *Mezilaurus crassiramea* (Meisn.) Taub. ex Mez., another lauraceous species commonly found in Mato Grosso do Sul state, Midwest Brazil [11,12]. These butanolides, which belong to the class of polyketide-derived γ-lactones, bear a long linear alkyl side chain, as well as one- or three- carbon substituents (either oxygenated or non-oxygenated) linked to the butyrolactone skeleton. In the case of compounds **1**–**3**, an additional hydroxyl functionality is found at the β position of the lactone ring.

Considering that few studies have reported the antitrypanosomal potential of this class of secondary metabolites, we evaluated the activity of five butanolides previously isolated by our group [10–12] from two lauraceous species, namely, *Aiouea trinervis* and *Mezilaurus crassiramea* (Lauraceae), against epimastigote forms of different strains as well as other evolutive forms of *T*. *cruzi*, after a treatment period of 4 h. We also evaluated the synergistic effect of active butanolides and studied the capacity of the most active butanolide to inhibit important mechanisms in the parasite's life cycle in both vertebrate and invertebrate hosts, emphasizing the ultrastructural study of the parasitic forms found in the vertebrate and the process of host cell infection.

## Materials and methods

### Compounds

The butanolides epilitsenolide C1 (**1**), epilitsenolide C2 (**2**), and isoobtusilactone A (**3**) were isolated from *Aiouea trinervis* Meisn. [10], while majoranolide (**4**) and rubrenolide (**5**) were obtained from *Mezilaurus crassiramea* (Meisn.) Taub. ex Mez [11,12]. License for research on Brazil’s biodiversity, number A7A7F7F.

### Epimastigote cultures of *T*. *cruzi* and cell viability assays

Epimastigote forms of three strains of *T*. *cruzi*, including Dm28c (TcI) [13], CL Brener (TcVI) [14], and Y strain (TcII) [15], were maintained in liver infusion tryptose (LIT) medium supplemented with 10% fetal bovine serum at 28°C [16] were kindly provided by Oswaldo Cruz Institute/Fiocruz. For the experiments, exponential phase cultures were used. All assays were performed in triplicates in 96-well plates.

The effect of the isolated compounds on the viability of epimastigote forms of Dm28c strain was determined by MTS [3-(4,5-dimethylthiazol-2-yl)-5-(3-carboxymethoxyphenyl)-2-(4-sulfophenyl)-2H-tetrazolium bromide] with PMS (5-methyl-phenazinium methyl sulfate) colorimetric assay, as described by Nunes et al. [10]. The parasite suspension was adjusted to a concentration of 10^6^ parasites/mL and treated with the compounds (50 μg/mL) for 72 h.

Next in later experiment, parasite suspension containing 10^7^ parasites/mL was treated for 4 h with 6 to 8 concentrations (12 to 250 μM for butanolides and 12 to 1,600 μM for benznidazole) of the compounds that showed activity at 50 μg/mL. Additionally, epimastigotes of CL Brener and Y strains were treated for 4 h with the butanolide that showed best overall results.

The absorbance of the formazan was measured at 490 nm using a microplate reader (Asys Expert Plus -Biochrom). In parallel, we determined the effect of 1% DMSO (negative control) and benznidazole (positive control) on the viability of epimastigotes and a replicate of each treatment was performed by fixing the epimastigotes with 4% paraformaldehyde prior to the addition of MTS/PMS (basal absorbance control).

### Cytotoxicity assay

Vero cells (CCL-81) isolated from the kidney of the African green monkey *Cercopithecus aethiops* [17] were purchased from ATCC® (Washington DC, USA). The cells were inoculated in 200 μL of Dulbecco’s modified Eagle (DMEM) medium in 96-well plates at a concentration of 2 × 10^4^ cells/well and incubated at 37°C for 24 h for adherence of the cells [18]. Six different concentrations of the compounds (12 to 900 μM for butanolides, and 200 to 1,600 μM for benznidazole) were added to the wells. After 4 or 24 h of treatment, the cells were washed and cell viability was assessed using MTT (3-(4,5-Dimethylthiazol-2-yl)-2,5-Diphenyltetrazolium Bromide) assay (2 mg/mL). After 4 h of incubation at 37°C in a CO_2_ atmosphere, the wells were washed and 100% DMSO was added to solubilize the formazan crystals. The absorbance was measured at 570 nm using an ELISA plate reader (Biotek Model EL-800, VT, USA). DMSO (1%) treatment served as negative control and benznidazole treatment served as positive control.

### Trypomastigote forms and lysis assay

Cell-derived trypomastigotes (Dm28c) were obtained from the supernatant of infected Vero cell cultures. Vero cells (9 × 10^5^ cells/mL) and trypomastigotes were seeded at a ratio of 1:10 into culture bottles (75 cm^2^) containing DMEM. After 4 h of incubation, the bottles were washed to remove unadhered trypomastigotes and incubated for another 96 h under CO_2_ atmosphere at 37°C. After 96 h of incubation, new trypomastigote forms were collected from the supernatant and used to perform the assays.

The lysis assay was performed in 96-well plates in technical triplicates (4 concentrations within the range 4–250 μM for butanolides and 200–1,600 μM for benznidazole), and the trypomastigotes (1 x 10^6^ parasites/well) were incubated in 200 μL of DMEM (supplemented with 10% FBS, 100 μg/mL streptomycin, and 100 IU/mL penicillin) with the compounds at 37°C for 4 h. The test, negative control (1% DMSO), and positive control (benznidazole) parasites were fixed and counted in Neubauer's chamber.

### Amastigote forms and antiproliferative assay

To determine the antiproliferative effect of butanolides on intracellular amastigote forms (Dm28c), Vero cells were used as host cells. Vero cells were cultured in DMEM and maintained in a CO_2_ atmosphere at 37°C.

Vero cells from cultures that showed greater than 70% confluency were added to 96-well plates at a concentration of 4 × 10^3^ cells/well along with trypomastigotes (ratio of Vero cells/trypomastigotes, 1:10). After 4 h of incubation for adherence and infection, the cells were washed with 1x phosphate buffered saline (PBS) and then incubated in DMEM for 20 h to allow differentiation of internalized trypomastigotes into amastigotes.

Vero cells infected with amastigotes were incubated for 4 or 24 h with the compounds (4 concentrations within the range 4–200 μM) in DMEM. After incubation, the cells were washed, fixed in methanol, stained with 4',6-Diamidino-2-phenylindole (DAPI), and photographed using Leica DMI 6000-B fluorescence microscope. The photos obtained were analyzed using the software ImageJ version 1.5 for quantification of total Vero cells and determination of the percentage of infected cells and number of amastigotes per cell. The 50% inhibitory concentration (IC_50_) was calculated using the infectivity index (II) described by Ceole et al. [19]. DMSO (1%) treatment served as negative control and benznidazole treatment served as positive control.

### Synergistic effect

The fixed-ratio method using combination index (Ci) and drug reduction index (DRI) was used to predict synergistic, additive, or antagonistic effects of butanolide combinations, as proposed by Chou and Talalay [20] and revised by same author [21–23].

Briefly, amastigote forms of Dm28c strain were treated for 24 h with four different concentrations (3–24 μM) of isoobtusilactone A and majoranolide, individually and in combination at the ratios 4:0, 3:1, 1:1, 1:3, and 0:4. After treatment, the host cells and amastigotes were stained with DAPI, quantified using Operetta CLS High Content Analysis System, and analyzed using the Harmony high-content imaging and analysis software version 4.5 [20]. IC_25_, IC_50_, IC_75_ and IC_90_ for each combination and each compound was calculated, with 1% DMSO treatment as negative control. Individual Ci values for each butanolide at the respective ratios were normalized and plotted in isobologram and drug reduction index plot.

### Infectivity assay

#### Treatment before infection

The trypomastigotes were treated with isoobtusilactone A (LC_50_/4 h of trypomastigotes) for 4 h, while the control trypomastigotes were incubated with drug-free medium for 4 h. These trypomastigotes were then seeded into 96-well plates (4 × 10^4^ parasites per well) with already adhered Vero cells at a ratio of 10 parasites per host cell.

After 4 h of incubation, the medium containing parasites that did not infect host cells was replaced with drug-free fresh medium. After 20 h of infection, the infected Vero cell culture with intracellular amastigotes was fixed, stained with DAPI, photographed, and analyzed as described for antiproliferative assay.

#### Treatment during infection

Already adhered Vero cells were incubated with recently collected trypomastigotes for 4 h in a medium containing isoobtusilactone A (LC_50_/4 h of amastigotes) or in a drug-free medium (negative control). After 4 h, the host cells were washed and drug-free fresh medium was added. The cells were incubated for another 20 h and then fixed, stained, photographed, and analyzed as described above.

### Inhibition of metacyclogenesis

During nutritional stress in triatomine artificial urine (TAU) medium, epimastigote forms of strain Dm28c were incubated with isoobtusilactone A (IC_50_/24 h of epimastigotes -2.75 μM, as described by Nunes et al. [10]) for 2 h. After the stress, the cells were washed and incubated with TAU drug-free supplemented with 10 mM L-proline, 50 mM L-sodium glutamate, 2 mM L-sodium aspartate and 10 mM D-glucose (TAU3AAG medium) for 72 h. The total number of differentiated trypomastigotes was counted in Neubauer's chamber to assess the inhibition percentage [24].

### Scanning electron microscopy

Trypomastigotes were incubated with isoobtusilactone A (LC_50_/4 h of trypomastigote) for 2 h. The parasites were washed with cacodylate buffer (0.1 M) and fixed in cacodylate buffer (0.1 M) containing paraformaldehyde (4%) and glutaraldehyde (2.5%). The cells were then adhered to glass cover slips previously coated with poly-L-lysine, postfixed with osmium tetroxide (1%), dehydrated with graded ethanol series, and dried using a critical point dryer (Leica CPD 300). Finally, the samples were coated with a layer of gold in a sputtering device (Leica EM ACE 200).

Similarly, intracellular amastigotes after 24 h of interaction with host cells were treated with isoobtusilactone A (LC_50_/4 h of amastigotes) for 4 h. The cells were fixed, dehydrated, and dried following the same procedure described above. Cell membrane of the host cells was randomly removed with the aid of an adhesive tape and the resulting samples were coated with a layer of gold in a sputtering device (Leica EM ACE 200).

For ultrastructural analysis of trypomastigote cells during the infection, trypomastigotes were treated as described for infectivity assay (**Treatment before infection**). After treatment, the parasites were seeded into 96-well plates (4 × 10^4^ parasites per well) with already adhered Vero cells at a ratio of 10 parasites per host cell. After 4 h of incubation, the host cells were washed and processed as described above.

All samples were analyzed using a scanning electron microscope (Jeol JSM-6010 Plus/LA) operating at 20 keV.

### Transmission electron microscopy

Trypomastigotes were treated following the same procedure as described for scanning microscopy. After treatment, the cells were washed with cacodylate buffer (0.1 M) and fixed in cacodylate buffer (0.1 M) containing paraformaldehyde (4%) and glutaraldehyde (2.5%).

Intracellular amastigotes and host cells, after 4 h of treatment with isoobtusilactone A (LC_50_/4 h of amastigotes), were detached by trypsinization, washed, and fixed in cacodylate buffer (0.1 M) containing paraformaldehyde (4%) and glutaraldehyde (2.5%).

For ultrastructural analysis of trypomastigote cells during infection, trypomastigotes were treated as described for infectivity assay (**Treatment before infection**) and processed as described above.

All samples were postfixed in osmium tetroxide (1%), dehydrated with acetone series, and embedded in EMbed 812® resin. Grids containing ultrathin sections (70 nm) were stained with uranyl acetate and lead citrate, and observed under a transmission electron microscope (Jeol 1,400 Plus) operating at 80 keV.

### Statistical analysis

The 50% cytotoxic concentration (CC_50_), x % inhibitory concentration (IC_x_ = IC_25_, IC_50_, IC_75_, or IC_90_), and 50% lethal concentration (LC_50_) values were obtained from the percentage of inhibition/death versus log concentration of each compound, by nonlinear regression with variable slope and automatic withdrawal of outliers with ROUT coefficient (Q = 5). Only regressions with coefficient of determination (R^2^) > 0.82 and standard error (SE) of log LC_50_, log CC_50_, or log IC_x_ lower than 0.11 were accepted; if the parameters were not met, a new experiment was performed. Graphpad Prism software version 7.04 was used for all regressions.

Statistical differences between treated and untreated groups were assessed using one-way analysis of variance (ANOVA) followed by Tukey's post hoc test. BioEstat software version 5.3 was used to perform ANOVA and Tukey’s test.

Selectivity index (SI), infectivity index (II), combination index (Ci), and drug reduction index (DRI) were calculated using Excel software version office 2016; the formulae used have been detailed in the following articles: SI [25]; II [19] and Ci and DRI [23]

## Results

### 1-Selection of the most active butanolides

**Fig 1:**
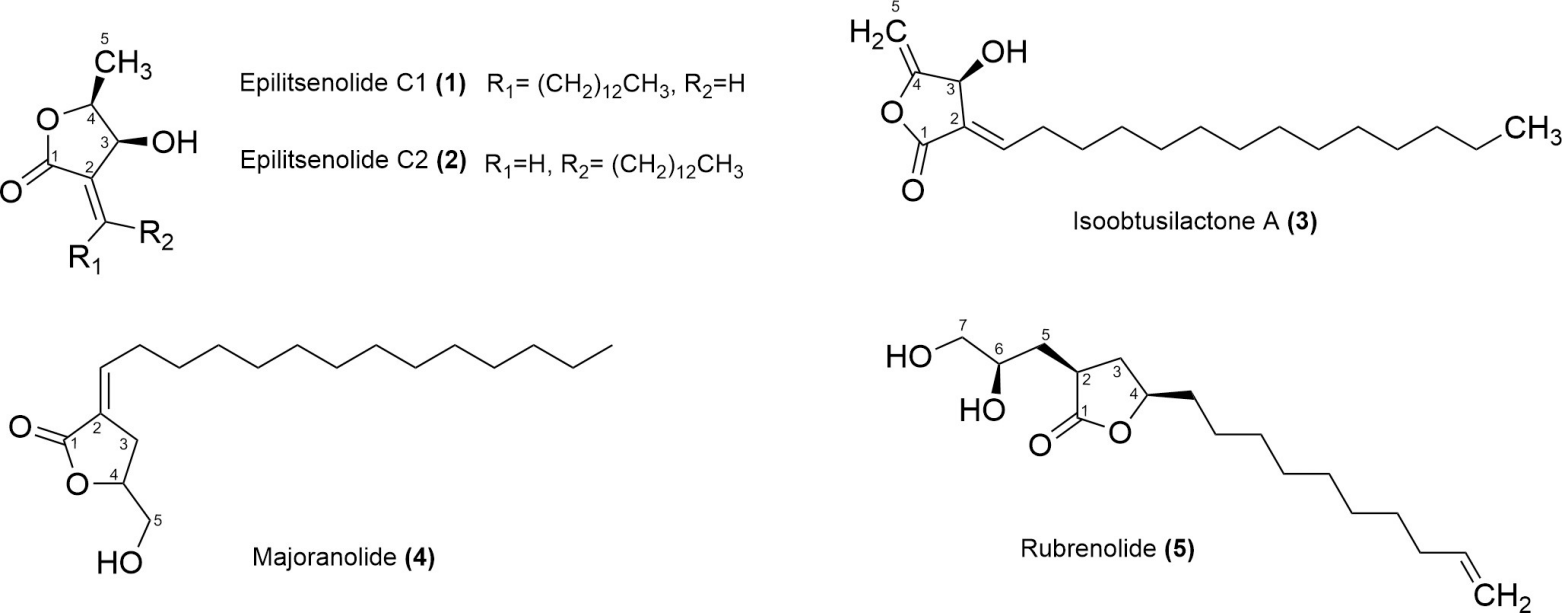
Chemical structures of the butanolides isolated from *Aiouea trinervis* (1–3) and *Mezilaurus crassiramea* (4 and 5).

Among the five butanolides evaluated (Fig 1), **1**, **3**, and **4** exhibited activity at concentrations below 50 μg/mL in epimastigotes after 72 h of treatment. However, only isoobtusilactone A (**3**) and majoranolide (**4**) were selected to continue the studies, since epilitsenolide C1 (**1**) did not show activity against epimastigotes when assessed in a short period of exposure (See item 3 of results).

### 2-Potential of butanolides against amastigotes alone or in combination

Both majoranolide and isoobtusilactone A exhibited similar activity against amastigote forms of the parasites after 24 h of treatment with the same pattern for selectivity indexes (Table 1). However, their activity did not exceed the activity of the control drug on amastigote forms.

**Table 1:** Activity of butanolides and benznidazole against amastigotes of *T*. *cruzi* in 24 hours of treatment.

Compounds	Amastigotes (Dm28c)*	Vero cells (CCL-81)*	SI
	IC_50_ (CI)	CC_50_ (CI)	
Isoobtusilactone A (**3**)	22.97 (16.1–33.9)	156.45 (144–169)^1^	6.81
Majoranolide (**4**)	21.92 (18.3–25.5)	166 (155–178)	7.57
Benznidazole	6.57 (5.3–7.8)	1145 (1100–1192)	174

Inhibitory concentration of 50% (IC_50_); Cytotoxic concentration of 50% (CC_50_); Selectivity index (SI); Confidence intervals (CI)

^1^Nunes et al., 2020

*Results expressed in micromolar (μM).

**Fig 2:**
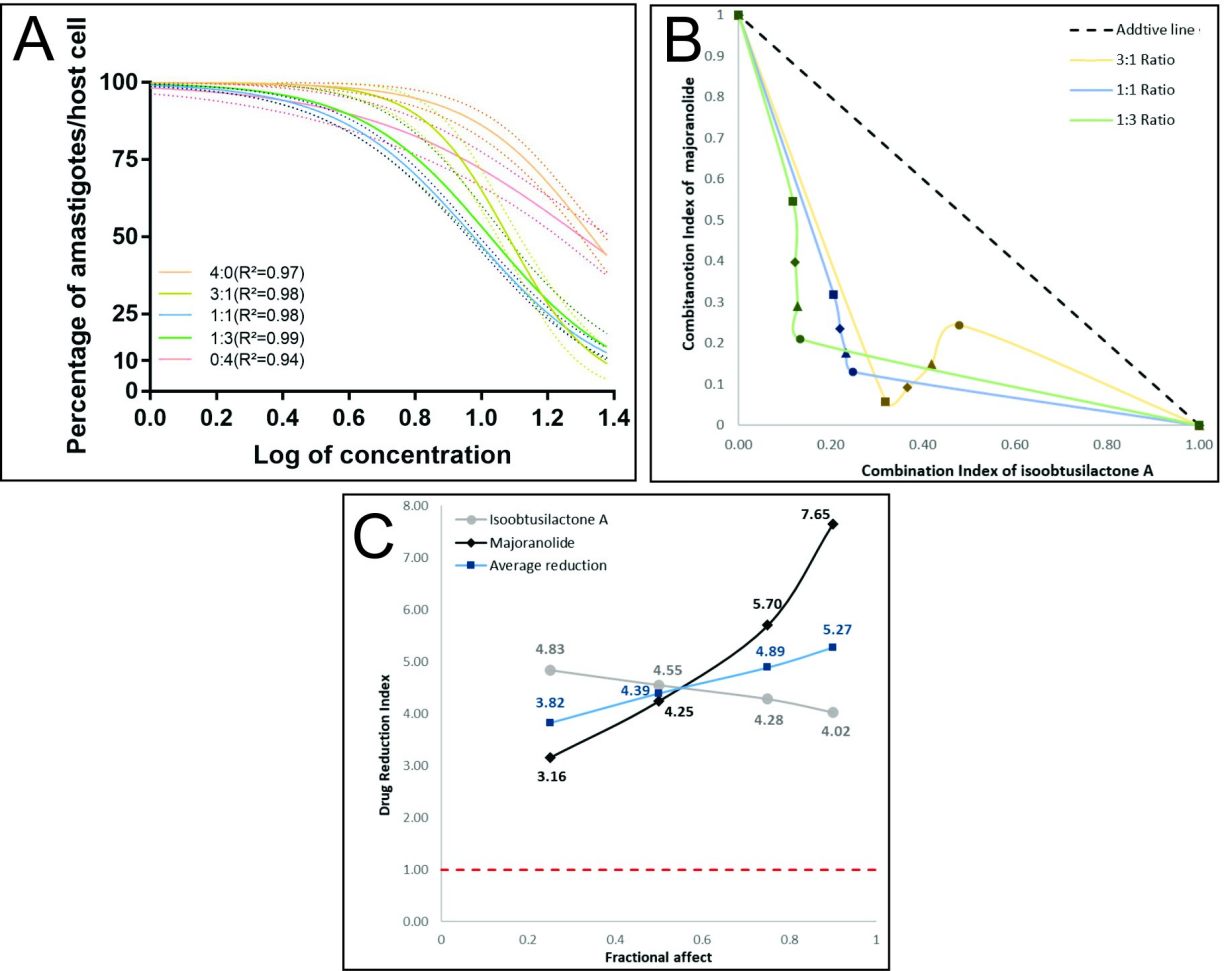
Synergism effect of isoobtusilactone A and majoranolide against amastigotes. A: Nonlinear regression with variable slope of each ratio (4:0-isoobtusilactone A alone; 0:4 majoranolide alone); B: Normalized isobologram of combination index of the ratios 3:1, 1:1 and 1:3 (■:IC_25_, ♦:IC_50_, ▲:IC_75_, ●:IC_90_); C: Drug reduction index plot (Dashed line represents the threshold of a favorable drug reduction).

The combination assay was used to improve activity and reduce toxicity with both active butanolides against amastigotes. For these reason, we choose to follow this synergism studies with an approach without the benznidazole, to bring new compounds insights, since synergistic assays with benznidazole have already been widely reported. These results are depicted in Fig 2.

To predict the effect of interaction between active butanolides on amastigote cells (Fig 2), the combination index (Ci) was determined. According to the theorem of Chou-Talalay, Ci = 1 indicates an additive effect, Ci < 1 indicates synergism, and Ci > 1 indicates antagonism, based on Ci, synergism can be further subclassified as: nearly additive (0.9–1), slight synergism (0.85–0.9), moderate synergism (0.7–0.85), synergism (0.3–0.7), strong synergism (0.1–0.3), and very strong synergism (< 0.1) [20,21].

The mean Ci of the butanolides **3** and **4** combination was 0.48, indicating synergism. This suggests that these butanolides can be used in combinations to improve their activity against amastigote forms of *T*. *cruzi* while reducing the dosage. The ratio 1:1, which demonstrates the best synergistic effect (Ci = 0.44), exhibited a better IC_50_ of 9.48 (4.74 μM of each compound); the DRI data indicated that the concentration of compounds required in this combination to obtain the same effect as that of individual drugs was up to 7 times less than the concentration of individual compounds. The selectivity index was also higher with this ratio as compared to individual drugs.

### 3-The fast action of butanolides

Among the three butanolides which were active after 72 h of treatment (**1**, **3**, and **4**), only isoobtusilactone A (**3)** and majoranolide (**4**) exhibited activity after 4 h of treatment, demonstrating a fast action (Table 2).

**Table 2:** Activity after a short period of treatment of butanolides and benznidazole against epimastigotes of *T*. *cruzi*.

Compounds	Epimastigotes (Dm28c)*	Vero cells (CCL-81)*	SI
	IC_50_ - 4h (CI)	CC_50_ - 4h (CI)	
Epilitsenolide C1 (**1**)	Na	Ne	Ne
Epilitsenolide C2 (**2**)	Na	Ne	Ne
Isoobtusilactone A (**3**)	10.09 (8.6–11.6)	371.08 (286–516)	36.7
Majoranolide (**4**)	590.96 (556–625)	557.74 (500–634)	0.94
Rubrenolide (**5**)	Na	Ne	Ne
Benznidazole	>1600	>1600	-

Inhibition concentration of 50% (IC_50_); Cytotoxic concentration of 50% (CC_50_); Selectivity index (SI); Confidence intervals (CI); Not evaluated (Ne); Not active (Na)

*Results expressed in micromolar (μM).

Both **3** and **4** showed better results than benznidazole against the epimastigote forms of strain Dm28c (TcI), but majoranolide (**4**) did not present good selectivity index in Vero cells, being more active against mammalian cells than protozoa. On the other hand, isoobtusilactone A (**3**) demonstrated better activity and high selectivity index (SI).

Corroborating the activity of these chemical compounds after 4 h, both isoobtusilactone A and majoranolide presented an effective trypanocidal activity against the trypomastigote forms of the parasite (Dm28c), with higher activity against the infective forms and better SI as compared to the control drug, which did not demonstrate activity into this time of exposure (LC_50_>1,600 μM). Isoobtusilactone A presented an LC_50_ of 30.05 μM (20.9–43.1) with an SI of 12.34, while majoranolide presented less activity with an LC_50_ of 71.67 μM (66.4–76.7) with an SI of 7.77.

It is recommended that a new drug candidate should be tested against different strains of DTUs to fit the characteristic complexity of the species and thus reduce the probability of failure in pre-clinical trials [26]. Therefore, we tested the activity of isoobtusilactone A, the most active butanolide against both trypomastigotes and epimastigotes, against the epimastigote forms of CL Brener (TcVI) and Y strains (TcII).

Isoobtusilactone A also demonstrated activity in these two strains, with IC_50_ of 32.28 μM (CI: 26.1–39.8; R^2^: 0.93) and 61.7 μM (CI: 51.4–74.0; R^2^: 0.91) and SI of 11.4 and 6.0 in strain Y and strain CL Brener, respectively. On the other hand, the control drug (benznidazole) presented the same pattern without activity (IC_50_> 1,600 μM) following the same time period of exposure in all three strains.

To further demonstrate the potential of isoobtusilactone A, we assessed the activity of isoobtusilactone A in comparison with benznidazole on amastigotes after a short period of exposure.

After a treatment period of 4 h, isoobtusilactone A maintained its high activity against amastigote forms (LC_50_ = 20.75 μM; CI = 13–32) with better SI (17.88), as compared to treatment period of 24 h. In contrast, at 4 h, the activity of benznidazole decreased by almost 13 times as compared to the activity at 24 h (LC_50_ = 81.59 μM; CI = 62–107), being about to four times less active than the butanolide.

### 4-Isoobtusilactone A induces mitochondria alterations

**Fig 3:**
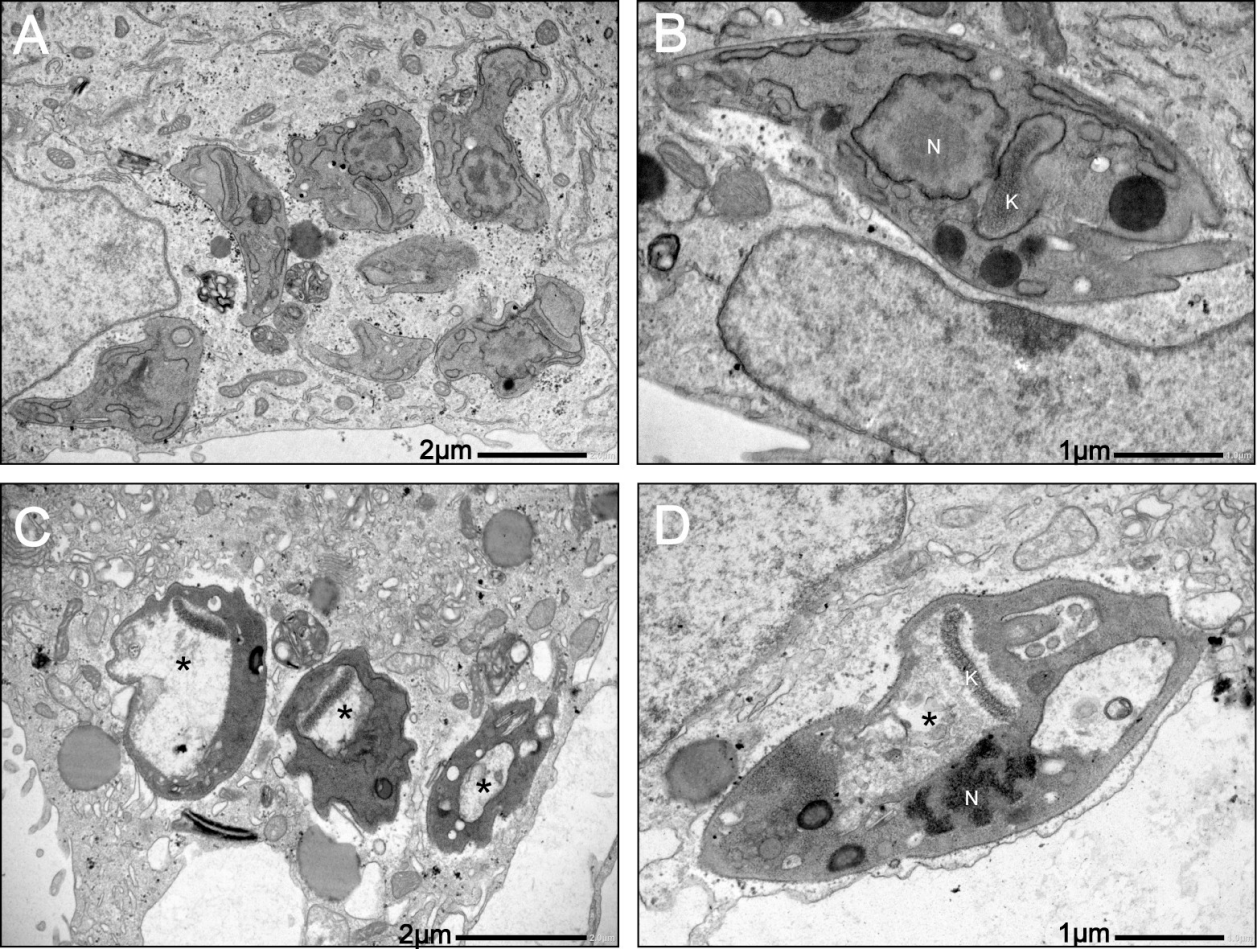
Transmission electron micrographs showing the ultrastructure of intracellular amastigotes. Nucleus (N); kinetoplast (K); *Swollen mitochondria. A-B: Control untreated; C-D: Parasites treated 4 hours with IC_50_ of isoobtusilactone A.

To better understand the mechanism of action of this butanolide in such a short period of time, the ultrastructure of treated amastigote cells was analyzed (Fig 3).

The treated amastigote cells demonstrated a strong and acute damage to the unique mitochondria of the parasite (Fig 3B), along with swelling and loss of internal electron-dense content, probably due to the loss of mitochondrial contents. These extensive organelle damages probably represent the main cause of parasite death. On the other hand, despite the intense damage to mitochondria, small alterations were observed in their external morphology (S1 Fig).

**Fig 4:**
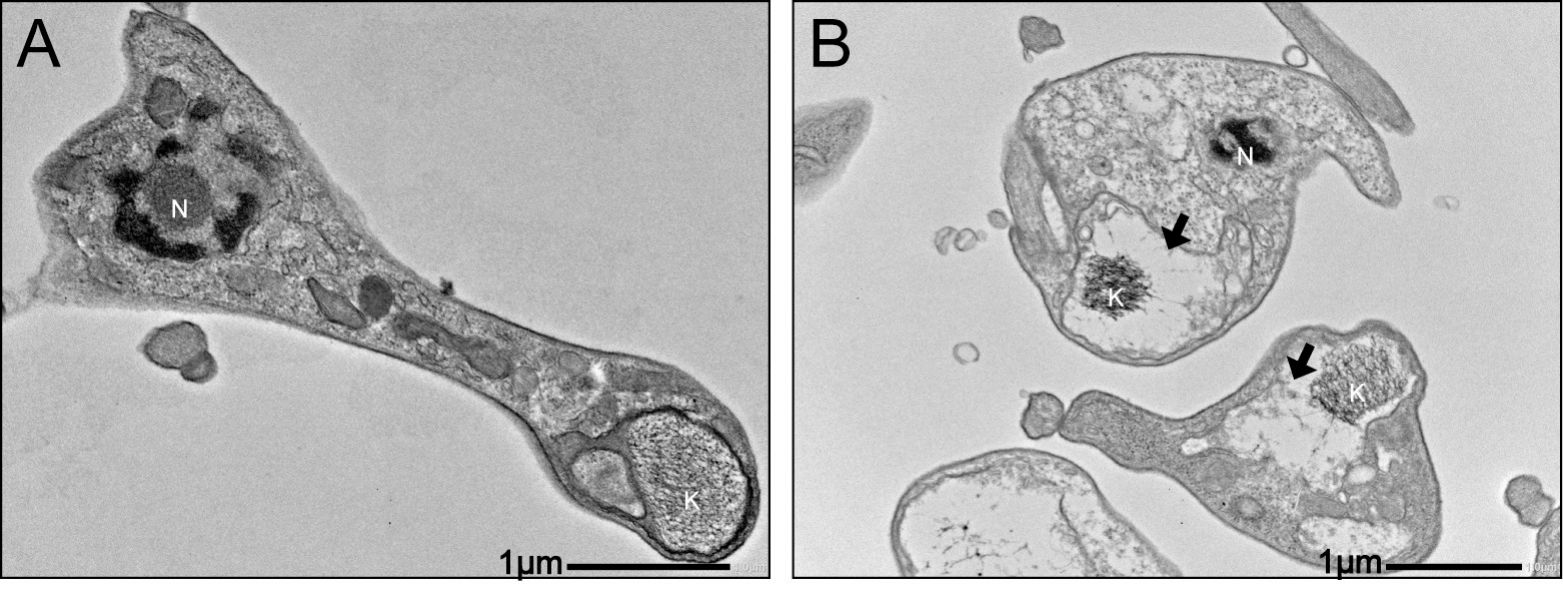
Transmission electron micrographs showing the ultrastructure of trypomastigotes. Nucleus (N); kinetoplast (K); swollen mitochondria (Black arrow). A: Control untreated; B: Treatment with isoobtusilactone A (LC_50_) for 2 hours.

To determine if mitochondrial damage plays the same pivotal role in the activity on the infective forms, the ultrastructure of trypomastigotes was also evaluated. The ultrastructural alterations were similar to that observed in treated amastigotes, with swelling and loss of internal content of mitochondria (Fig 4), reinforcing that mitochondrial damages are intrinsically related to the trypanocidal activity of this compound.

**Fig 5:**
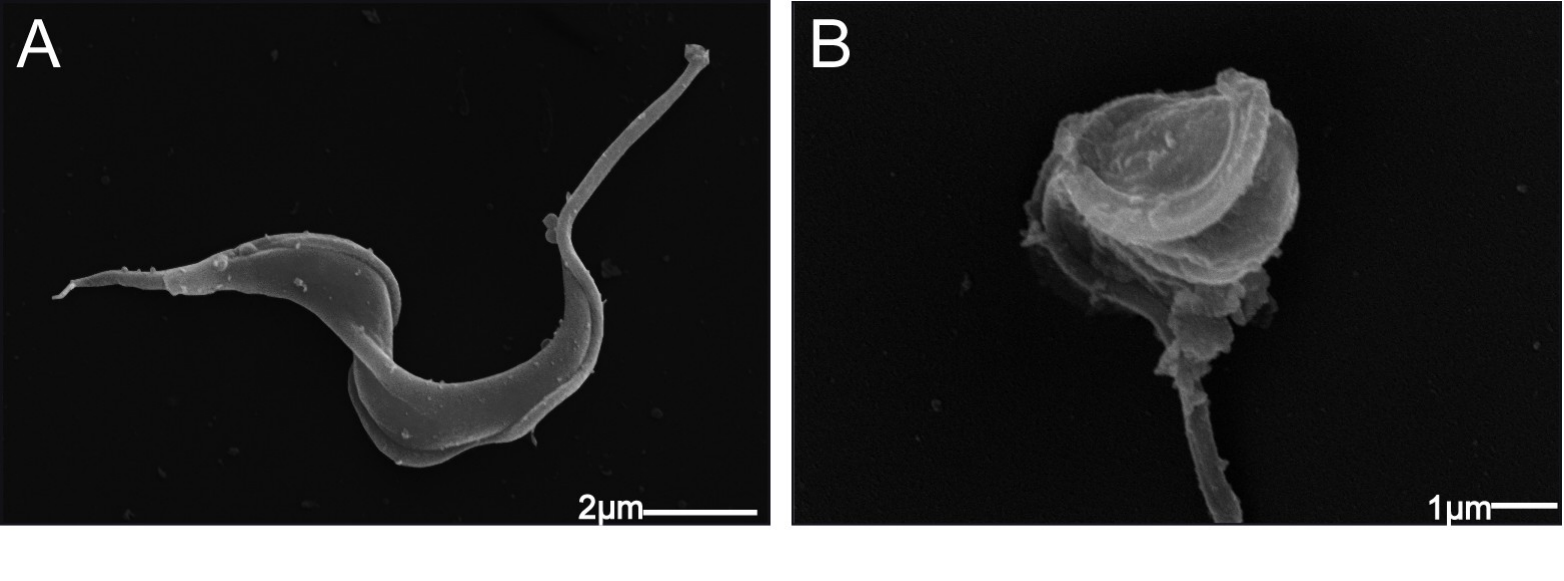
Scanning electron micrographs showing the morphology of trypomastigotes. A: Control untreated; B: Treatment with isoobtusilactone A (LC_50_) for 2 hours.

The prominent damages to the mitochondria were followed by a drastic change in the morphology of the infective forms (Fig 5).

The morphology of the treated trypomastigotes was highly altered, with loss or shrinkage of the flagellum and spherical body shape, which leads to a reduction in the mobility of the parasite (observed under an optical microscope). These alterations could reduce the capacity of these parasites to invade new host cells as well as the capacity to find new cells to infect.

### 5-Isoobtusilactone A interferes life events of the parasite

To reinforce the promising potential of isoobtusilactone A, crucial events in the parasite's life cycle were evaluated, since molecules capable of altering these pathways are considered as interesting targets.

Based on the changes observed on trypomastigotes after a short period of treatment with isoobtusilactone A, such as the shrinkage or loss of the flagellum and the spherical body shape, we investigated the ability of this butanolide to interfere with the host cell invasion, as well as the differentiation of the parasite, both of which are essential for the success of the pathogen in the host.

The metacyclogenesis inhibition assay demonstrated that isoobtusilactone A (IC_50_/24 h of epimastigotes) inhibited the differentiation of epimastigotes *in vitro*. After 72 h of incubation in a drug-free medium, the treated epimastigotes presented 43% less trypomastigotes than control (p = 0.009). These results indicate that after a short period of exposure (2 h) with isoobtusilactone A at a concentration of 2.75 μM the metacyclogenesis *in vitro* was inhibited.

**Fig 6:**
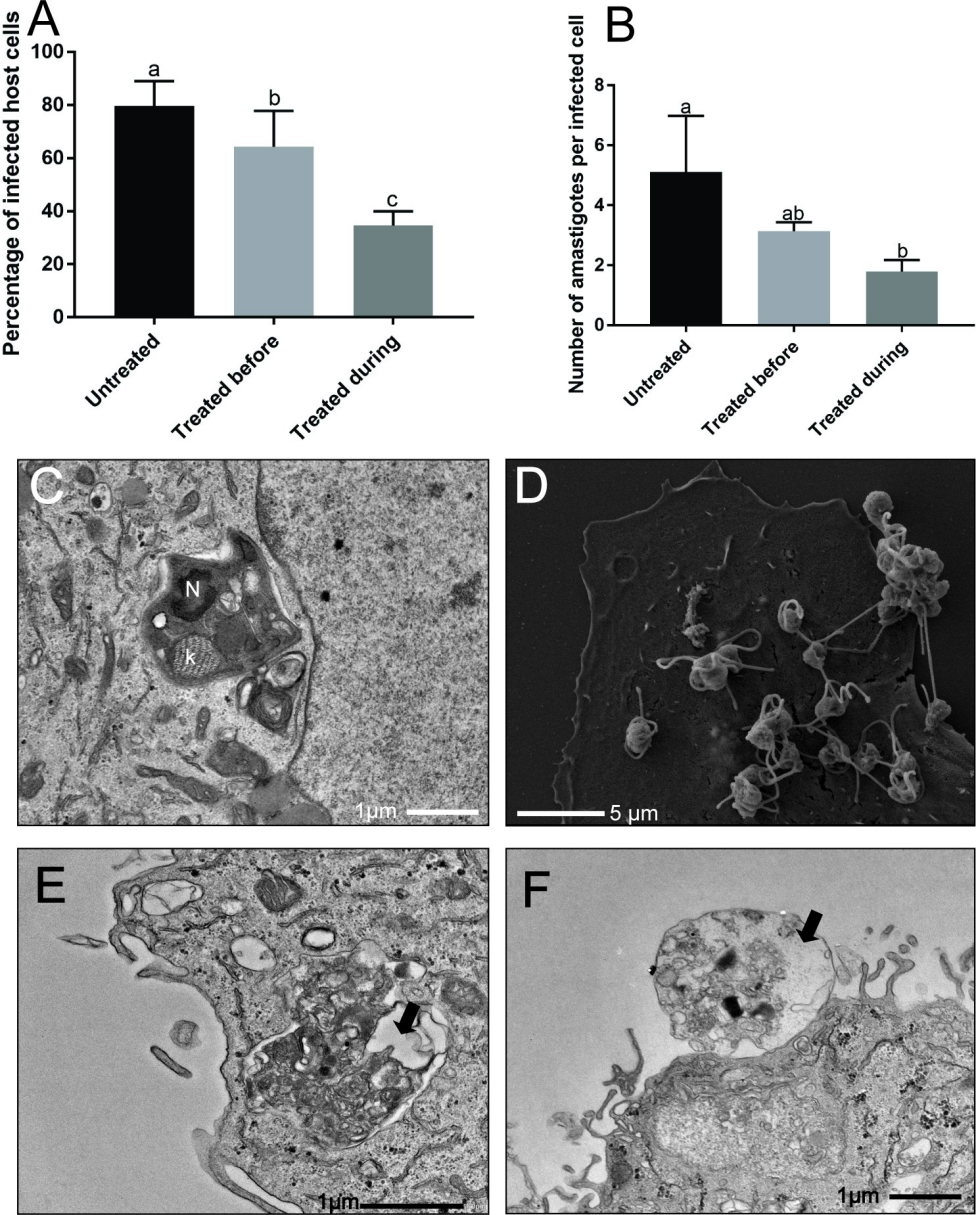
Evaluation of the interference of isoobtusilactone A on infection of host cells. Nucleus (N); kinetoplast (K); intense cytoplasm disorganization (Black arrow). A: Percentage of infected cells by trypomastigotes (p<0.05 by Tukey); B: Number of amastigotes per infected cells (p<0.05 by Tukey); C: Transmission electron micrographs showing the morphology of infected cells with untreated trypomastigotes; D: Scanning electron micrographs showing the morphology of host cells after infection with treated trypomastigotes; E-F: Transmission electron micrographs showing the morphology of infected cells with treated trypomastigotes.

The trypomastigotes treated with isoobtusilactone A (LC_50_/4 h of trypomastigote) before infection exhibited statistic less capacity to infect new host cells (Fig 6), with 20% reduction in the number of infected cells and almost 40% reduction in the number of amastigotes per cell. On the contrary, treatment simultaneous to the infection with lower concentration of the drug (LC_50_/4 h of amastigote) demonstrated better results, with 43% reduction in the number of infected cells and 65% reduction in the number of amastigotes per cell (Fig 6A and 6B).

To better understand the mechanism by which the infectious ability of trypomastigotes is impaired by this butanolide, we studied the ultrastructure of host cells and treated trypomastigotes after 4 h of interaction.

In control host cells infected with untreated trypomastigotes, after 4 h of interaction, all trypomastigotes were internalized and some already escaped the parasitophorous vacuole (Fig 6C). In contrast, in host cells infected with treated trypomastigotes, the majority of the parasites adhered to host cells (Fig 6D) and only fewer parasites were internalized (Fig 6E).

The morphology of the trypomastigote forms and the high number of parasites adhered to host cells (Fig 6D), similar with the morphology of trypomastigotes observed after 2 h of treatment with isoobtusilactone A (Fig 5B), indicate that parasites with spherical shape and damaged flagellum were unable to internalize. Although some parasites were capable of infecting host cells, the majority of treated parasites already showed ultrastructural damage with cytoplasmic disorganization (Fig 6E and 6F), which could also further interfere with the infectious capacity of these trypomastigotes. Another alteration observed in treated trypomastigotes was wrinkling of the protozoan surface (cell membrane).

## Discussion

It has already been reported that **1** and **3** demonstrated activity after 24 h of treatment against epimastigote forms of Dm28c strain, with IC_50_ of 7.77 and 2.75 μM, respectively [10]. However, in the present study, we evaluated their activity after 4 h of treatment and demonstrated that, between the two butanolides, isoobtusilactone A presented a faster action, with almost the same activity reported at 24 h.

In our previous study [10], it was postulated that the presence of a Δ^4,5^ unsaturation and the conjugated double bond in the α-alkylidene-γ-lactone skeleton were important structural requirements for the activity of butanolides against *T*. *cruzi* epimastigotes after 24 h of treatment. In another study [9], it was shown that the butanolides isolinderanolides D and E, also bearing an exocyclic double bond functionality at C-4 and a C-2/C-6 conjugated double bond, were active against trypomastigotes and amastigotes, respectively. Based on the results obtained for **3** and **4** in the present study (Table 2), both the presence of an exocyclic double bond at C-4 and a hydroxyl functionality situated β to the lactone carbonyl in **3** seem to play a key role in the effect of this butanolide against epimastigotes, since the presence instead of a hydroxymethylene function at C-4 and the absence of the hydroxyl group at C-3, as found in **4**, led to an expressive reduction of activity, as well as in the SI value. Accordingly, butanolide **5**, a non-conjugated butanolide, bearing a di-hydroxylated three-carbon chain at C-2 and a linear unsaturated alkyl side chain at C-4, was devoid of any activity against epimastigotes.

Other plant constituents bearing a γ-lactone core, namely sesquiterpene lactones and lignan lactones, have also presented significant results against epimastigote forms of *T*. *cruzi* [27–32]. The antitrypanosomal activities of some sesquiterpene lactones, as well as their cytotoxic properties have been associated with the presence of a potentially reactive α-methylene-γ-lactone moiety that can acts as a Michael acceptor for biological nucleophiles, although other structural features, including stereochemical arrangements and hydrophobicity, seem to play key roles in determining their bioactivities [32–35]. Apart from the presence of a γ-lactone backbone, the foregoing classes of secondary metabolites, which originate from diverse biosynthetic pathways from that of the lauraceous butanolides [36], bear no other structural resemblance to isoobtusilactone A, as well as to the other butanolides **1**, **2**, **4**, and **5** reported in the present study.

Compounds **1**–**4**, despite their potential as Michael acceptors by the presence of a conjugated α-alkylidene side chain at C-2, possess other structural features that must influence their antitrypanosomal activity, given the highest potency of isoobtusilactone A (**3**) against epimastigotes after a 4 h treatment, when compared to those of the other three butanolides that also bear the same conjugated 14-carbon side chain. Therefore, the influence of the stereochemistry at C-2 in **3** and **4** (*E*) and (*Z*), respectively, as well as the already mentioned nature of the substituents at C-3 and C-4 on the activity of these butanolides must also be considered.

In addition, much attention should be given when comparing the reported antitrypanosomal activities of some sesquiterpene and lignan lactones to those of the butanolides, due to other important aspects of those studies, such as the period of exposure to the plant constituents and the type of strain used. In previous studies, for example, the activity of some of the former compounds against epimastigotes were evaluated after 24 or 72 h of treatment [27–30]. In contrast, in the present study, the activity of isoobtusilactone A against epimastigotes forms of *T*. *cruzi* (Dm28c strain) was evaluated after 4 h of treatment and a high SI was observed. This indicates the relevance of our study with the butanolide isoobtusilactone A, as there are no reports on lactones exhibiting activity in such a short period of exposure.

According to the preceding data, isoobtusilactone A was active on epimastigotes of the three tested strains, in a short treatment *in vitro*, being more active against Dm28c strain. This was one of the reasons to select this butanolide to continue our study. It is known that the susceptibility depends on several aspects related to each strain, as well as on host interactions [37,38], but this aspect could not be discussed here due to the need of *in vivo* studies.

The Drugs for Neglected Diseases initiative (DNDi) suggests that a candidate drug for *in vivo* trials should have an SI greater than 10 [26]. Isoobtusilactone A exhibited an SI greater than 10 against the strains Y and Dm 28c, which reaffirms the biological potential of butanolides as anti–*T*. *cruzi* agents.

In contrast to their activities against epimastigotes at 4 h (Table 2), wherein isoobtusilactone A (**3**) proved more active than majoranolide (**4**) by roughly 59-fold, with an SI value about 39-fold higher, the activity of **3** against trypomastigotes at 4h was only about 2.4 higher than that of **4**, with almost comparable selectivity indexes. Therefore, structural features other than those mentioned for the activity of **3** and **4** against epimastigotes might be responsible for the activity of these butanolides against trypomastigote forms.

Study by Conserva et al. [9], demonstrated that the butanolides isolinderanolide D and isolinderanolide E, and the secobutanolide secosubamolide A exhibited high antitrypanosomal activity against trypomastigotes, with IC_50_ of 12.9 μM, 29.9 μM, and 12.5 μM, respectively, however, the period of treatment was 24 h. The authors also demonstrated similar activity of isolinderanolide D (25.3 μM), isolinderanolide E (10.1 μM) and secosubamolide A (12.3 μM) against amastigote forms, although with longer treatment periods.

Certain sesquiterpene and lignan lactones were also more active than isoobtusilactone A, against amastigote and trypomastigote forms [28–32], but mostly presented high toxicity, moreover the long exposure period could overestimate the results.

A molecule to be considered promising must have, in addition to a high activity against the target and a low toxicity, other characteristics that indicate it as an interesting hit to be evaluated more deeply, especially when considering the extensive list of active compounds against *T*. *cruzi* available in the literature [39–41].

For this purpose, we seek in this work to select a butanolide that in addition to having a high activity against the parasite and a low toxicity, presents a fast action, deficient in the prodrug currently available on the market, benznidazole [19,42,43].

Short exposure periods have been explored by researchers to identify potential compounds with a fast mechanism of action, which is a preferential attribute of candidate drugs. In this scope, there are few reports in the literature on compounds that present these characteristics [19,26,44–46]. Thus, isoobtusilactone A and majoranolide are potential drug candidates for treatment of *T*. *cruzi* infections.

The high activity of both the butanolides, in a short period of exposure, reinforce that these compounds can be more effective in affecting both circulating trypomastigotes and intracellular amastigotes.

As expected, the reference drug, benznidazole, did not present good results with short periods of exposure *in vitro*, due to the need for biotransformation to exhibit biological activity [42]. This fact corroborates the inability of this drug to combat trypomastigotes after a short period of exposure [19,43].

On the other hand, with amastigote forms, our data from *in vitro* treatment with benznidazole revealed its high activity and low toxicity, which is the same pattern found by other authors [19,47]. Despite the promising results *in vitro* for benznidazole, this drug maintains the high rate of side effects and the low efficacy reported in clinical studies [48,49].

Authors have sought to improve the clinical effects of benznidazole with various combinations, both with other drugs already approved [41,50–54], and with other natural compounds that showed anti-*T*. *cruzi* activity *in vitro* [55,56].

Associations with benznidazole have not always represented a promising alternative, showing antagonism or only an additive effect among the drugs tested [56–58]. Although some combinations with benznidazole have shown good results in *in vitro* assays [55,59,60], few have led to clinical studies and also with unsatisfactory results [41,52]. These data could justify our choice of compounds for combination tests excluding benznidazole, as already reported in the literature [61–63].

It is worth mentioning that the combination of the butanolides isoobtusilactone A and majoranolide demonstrated a synergistic effect with less toxicity, increased activity and savings in the amount of drugs needed to achieve the same effect. It is also noteworthy that the synergistic effect was observed in other proportions evaluated for the combination of butanolides.

Majoranolide exhibited good results against the amastigote form in 24 h, but it did not show the same effect against the different forms of the parasite, such as low activity in the epimastigote form. On the other hand, isoobtusilactone A demonstrated strong activity with little variation between forms (10 to 31 μM) and also against different strains (10 to 62 μM). This conserved activity in conjunction with similar ultrastructural changes in the different forms of the parasite, indicates that possibly the target of action of isoobtusilactone A does not present relevant variation among the forms and strains of *T*. *cruzi*.

There are several experimental strategies to discover new compounds for the treatment of Chagas disease. Regarding *in vitro* drug testing, there is a consensus that different strains should be used for screening and that the selected compounds should be tested at different stages in the parasite's life cycle [64]. In this respect, isoobtusilactone A has proved to be the most promising butanolide so far in the literature, since it maintained the activity regardless of the parasite form or strain, and also affected essential events for the parasite's life cycle.

Other similar studies have demonstrated the inhibitory capacity of different compounds on *in vitro* differentiation of *T*. *cruzi*. Citral, a terpenoid constituent of the essential oil of *Cymbopogon citratus*, was capable of inhibiting metacyclogenesis in 54% at 30 μg/mL [65]. Souza et al. [66] showed that glutamine analogues inhibited the differentiation process at concentrations close to 6 μM for 144 h of treatment. Likewise, a calpain inhibitor, at a concentration of 50 μM in a 96-hour treatment also inhibited differentiation by almost 50% [67], which represents a longer exposure time than that used in the present study.

Also, histone deacetylases inhibitors in higher concentrations than isoobtusilactone A were able to reduce the amount of trypomastigotes by approximately 20% after treatment. These inhibitors were, similarly to isoobtusilactone A, capable of altering the infection of host cells after treatment of trypomastigotes. However, isoobtusilactone A led to a reduction in infectious capacity, while histone deacetylases inhibitors led to an increase in the infectivity, without causing changes in amastigotes [68].

The inhibition data obtained for isoobtusilactone A were promising, since in low concentration (2.75 μM) and only 2 h of treatment this compound was able to inhibit 43% of differentiation.

The effect on metacyclogenesis can be attributed to two factors: the ability of isoobtusilactone A to prevent epimastigotes adhesion, a prerequisite event for differentiation, or its direct interference in factors that trigger metacyclogenesis [65,69]. In infectivity assays, it was observed that the adhesion of the trypomastigotes in the host cell was not impaired (Fig 6D), which may suggest that the activity of isoobtusilactone A could be due to direct interference with some stages of the differentiation mechanism.

Regarding the infectivity assay, it was observed that this butanolide reduced 43% of the number of infected cells and 65% of the number of amastigotes per cell with a treatment of 20.75 μM for 4 hours.

In a similar study of infective capacity, the calpain inhibitor MDL28170, demonstrated an inhibitory effect when the trypomastigotes were treated for 1 h before the infection, reducing it from 20% to 50% according to the increase in concentration up to 50 μM [70]. The polyphenol resveratrol was also able to reduce the infection by half when at a concentration greater than 40 μM, but only using an exposure time of 18 hours [68].

According to Quilles et al. [71] cysteine proteases inhibitors used at 10 μM to treat trypomastigotes for 2 h were able to inhibit between 30 to 70% of the infective capacity. Two of these inhibitors, as in our study, showed that the treatment of trypomastigote forms during the infectious process caused greater inhibition than the treatment before the infection. However, the authors used 18 h of interaction, while we used only 4 h.

In our study, the high number of trypomastigotes on the membrane of host cells (Fig 6D) and the low overall number of amastigotes after differentiation (Fig 6B) indicate that isoobtusilactone A treatment resulted in defective or delayed infection, mainly impairing the internalization event.

In the present study, another alteration observed in treated trypomastigotes was wrinkling of the protozoan surface (cell membrane). Since it has been reported that a healthy membrane is important for successful infection by parasites [72,73]; the alteration of protozoan cell membrane might affect the internalization event, leading to fewer infected cells and amastigotes. Moreover, the extensive cellular disorganization observed in some treated trypomastigotes during the internalization process (Fig 6E and 6F) and the intense mitochondrial damage in infective forms (Fig 4B) could lead to death of the parasite, resulting in lower number of infected cells and amastigotes.

Alterations in the mitochondria are common in parasites treated with natural compounds [74–76]. Loss of potential of the mitochondrial membrane was observed in trypomastigote form of *T*. *cruzi* Y strain treated with other butanolides, which suggested that these changes could represent an early event in the mechanism of death, such as apoptosis or necrosis [9]. Although in a completely different experimental model, isoobtusilactone A showed activity against tumor cells, inducing apoptosis in Hep G2 cells [77–79]. The ultrastructural changes observed in the mitochondria of trypomastigote and amastigote forms may indicate that treatment with isoobtusilactone A can also lead to the loss of the potential of the mitochondrial membrane, but these changes cannot be verified in transmission electron microscopy.

In conclusion, our results demonstrated that a short period of treatment with the butanolides isoobtusilactone A and majoranolide could inhibit *T*. *cruzi*, suggesting the importance and potential use of these compounds, alone or in combination due to its promising synergistic effect and fast action. Isoobtusilactone A also affects different DTUs and all life forms of the parasite, with low concentrations without presenting toxicity to the host cells. Another relevant feature of this butanolide was its ability to cause major damage to mitochondria, in amastigote and trypomastigote forms of the parasite. Furthermore, this compound was able to inhibit both metacyclogenesis and infection of new host cells immediately after exposure to the drug, indicating that this compound might be an important alternative to the currently available drugs.

## Supporting information

S1 Fig Scanning electron micrographs showing the morphology of intracellular amastigotes. A: Control untreated; B: Treatment with isoobtusilactone A (IC_50_) for 4 hours.
(TIF)
