# Supplementary figures and images for: Synergistic effect and ultrastructural changes in Trypanosoma cruzi caused by isoobtusilactone A in short exposure of time
Source: PLoS One. 2021 Jan 28;16(1):e0245882. doi: 10.1371/journal.pone.0245882 (PMC7842926; doi:10.1371/journal.pone.0245882)

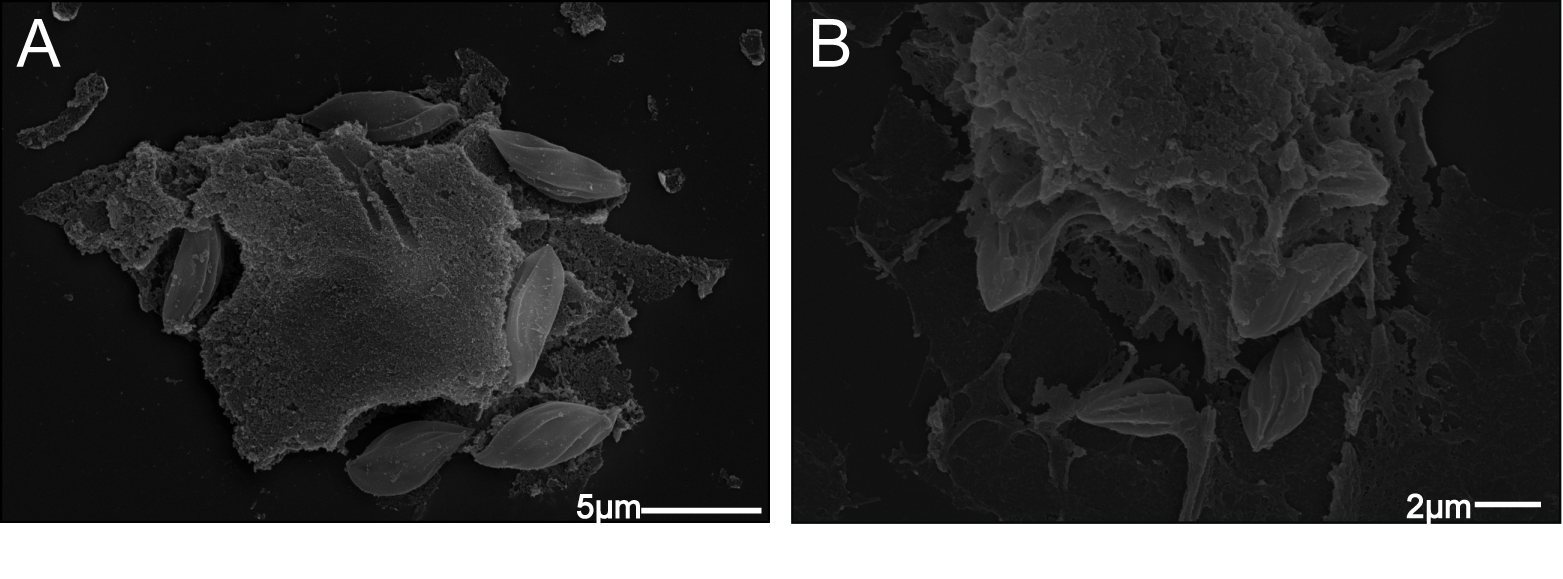

Supplement: S1 Fig — A: Control untreated; B: Treatment with isoobtusilactone A (IC50) for 4 hours. (TIF) [file pone.0245882.s001.tif]
